# The *Vibrio cholerae* Extracellular Chitinase ChiA2 Is Important for Survival and Pathogenesis in the Host Intestine

**DOI:** 10.1371/journal.pone.0103119

**Published:** 2014-09-22

**Authors:** Moumita Mondal, Dhrubajyoti Nag, Hemanta Koley, Dhira Rani Saha, Nabendu Sekhar Chatterjee

**Affiliations:** 1 Division of Biochemistry, National Institute of Cholera and Enteric Diseases, Kolkata, India; 2 Division of Bacteriology, National Institute of Cholera and Enteric Diseases, Kolkata, India; 3 Division of Electron Microscopy, National Institute of Cholera and Enteric Diseases, Kolkata, India; University of Illinois at Chicago College of Medicine, United States of America

## Abstract

In aquatic environments, *Vibrio cholerae* colonizes mainly on the chitinous surface of copepods and utilizes chitin as the sole carbon and nitrogen source. Of the two extracellular chitinases essential for chitin utilization, the expression of *chiA2* is maximally up-regulated in host intestine. Recent studies indicate that several bacterial chitinases may be involved in host pathogenesis. However, the role of *V. cholerae* chitinases in host infection is not yet known. In this study, we provide evidence to show that ChiA2 is important for *V. cholerae* survival in intestine as well as in pathogenesis. We demonstrate that ChiA2 de-glycosylates mucin and releases reducing sugars like GlcNAc and its oligomers. Deglycosylation of mucin corroborated with reduced uptake of alcian blue stain by ChiA2 treated mucin. Next, we show that *V. cholerae* could utilize mucin as a nutrient source. In comparison to the wild type strain, Δ*chiA2* mutant was 60-fold less efficient in growth in mucin supplemented minimal media and was also ∼6-fold less competent to survive when grown in the presence of mucin-secreting human intestinal HT29 epithelial cells. Similar results were also obtained when the strains were infected in mice intestine. Infection with the Δ*chiA2* mutant caused ∼50-fold less fluid accumulation in infant mice as well as in rabbit ileal loop compared to the wild type strain. To see if the difference in survival of the Δ*chiA2* mutant and wild type *V. cholerae* was due to reduced adhesion of the mutant, we monitored binding of the strains on HT29 cells. The initial binding of the wild type and mutant strain was similar. Collectively these data suggest that ChiA2 secreted by *V. cholerae* in the intestine hydrolyzed intestinal mucin to release GlcNAc, and the released sugar is successfully utilized by *V. cholerae* for growth and survival in the host intestine.

## Introduction

*Vibrio cholerae* is a Gram negative water-borne pathogen that causes the severe dehydrating diarrhea, cholera, which may turn fatal in absence of timely intervention by rehydration therapy. Outside human host *V. cholerae* is a normal resident of aquatic environment, where it colonizes on the chitinous surface of crustaceans [1]. Chitin the un-branched long chain polymer of β-1, 4 linked N-acetylglucosamine residues (GlcNAc) and is the main structural polysaccharide of the crustaceans. In addition to providing a safe niche to the marine bacteria, chitin also serves as the sole source of carbon and nitrogen in the nutritionally poor aquatic environment [1]. For successful utilization of chitin, marine bacteria including *V. cholerae* require a group of enzymes called chitinases and chitin binding proteins (CBP). These enzymes catabolically convert crustacean chitin into biologically useful molecules by hydrolyzing the β-1, 4 linkages between the GlcNAc residues, yielding soluble GlcNAc oligomers. *V. cholerae* primarily use two extracellular chitinases, ChiA1 and ChiA2 [2] that synergistically hydrolyze chitin into small oligomers [3]. Reports from other laboratories have indicated that environmental factors have a significant effect on expression and activity of these chitinase genes [1], [4]. Chitin utilization by *V. cholerae* is a complex process including steps such as chitin sensing, attachment and degradation. The two extracellular chitinases, ChiA1 and ChiA2 play an important role in initialization of *V. cholerae* chitin catabolic cascade [3]. When *V. cholerae* comes in contact with chitin, these enzymes play a collective but differential activity to degrade chitin into (GlcNAc)_n>2_ oligosaccharides [5], [6] that are transported into the periplasmic space via chito-porin [7]. However, the smaller molecules like GlcNAc monomer and the dimer N, N′-diacetylchitobiose enter the periplasm by nonspecific porins. In the periplasm, chitodextrinases and β-N-acetylglucosaminidases degrade chito-oligosaccharides into GlcNAc monomer and dimer which are transported through the inner membrane and enter the cytoplasm [3].

Although it is unclear how chitinases and chitin binding protein can promote pathogenesis in animal hosts with no apparent chitinous surface, there are reports that do indicate the involvement of bacterial chitinases and CBP in virulence [8], [9]. The two chitinases (ChiA and ChiB) and a CBP of *Listeria monocytogenes* are important in infection of liver cells [10]. ChiA is involved in bacterial survival, presumably by modulation of host immune response [11] whereas the ChiB and CBP may play a role in the initial colonization of the host cells. *Legionella pneumophila* contains a chitinase (ChiA) and a CBP. The ChiA had no effect on bacterial growth in an intracellular cell culture, but it enhanced bacterial survival in mammalian host cells [12]. ChiA of adherent-invasive *Escherichia coli* (AIEC strain LF82) plays a role as a virulence-factor, and may promote bacterial adhesion to mucosal tissues enhancing the pathogenic effects of AIEC [13]. CBP and a chitinase were also found in some clinical isolates of *Pseudomonas aeruginosa*. It has been suggested that *P. aeruginosa* utilizes CBP to promote adhesion of bacteria to lung epithelial cells during the initial phases of pulmonary infections in cystic fibrosis patients. Deletion of *V. cholerae* CBP, also known as GbpA resulted in reduced adherence to chitin and also to human intestinal epithelial cells *in vitro* which indicated it plays a key role in adhering to the chitinous exoskeleton of zooplanktons during chitin utilization as well as the human gastro-intestinal tract [14]. Subsequent binding studies showed that *V. cholerae* adheres to the intestinal epithelial cells through a coordinated interaction of GbpA with mucin [15]. Mucin, the large extracellular glycoprotein found on the surface of intestinal epithelial cells is highly glycosylated with ∼80% carbohydrates consisting primarily of N-acetylglucosamine, N-acetylgalactosamine, fucose, galactose and sialic acid and traces of mannose and sulphated sugars. Twenty percent of its molecular mass is constituted by the peptide backbone comprised of a large number of tandem repeats rich in serine, threonine and proline [16]. It is not clear how chitinases with their β-1, 4 glycosidase activity can modify mucin.

Although *V. cholerae* CBP plays a role in virulence, not much is known about the role of *V. cholerae* chitinases in pathogenesis. Since *V. cholerae* chitinases are constitutively expressed in the intestinal environment, it is possible that these chitinases may act as virulence factors. However, studies were directed towards better understanding of their natural chitinolytic role in the environmental context and not in terms of virulence. Here, we show that ChiA2, a *V. cholerae* extracellular chitinase, is involved in virulence. The enzyme helped *V. cholerae* to utilize mucin as a nutrient source for better survival in the host intestine. The survival of the Δ*chiA2* mutant was compromised in the intestinal environment and thus they demonstrated reduced virulence in animal models. This is the first indication to show that *V. cholerae* chitinase ChiA2 might promote the bacterial survival and virulence in host.

## Materials and Methods

### Ethics Statement

All animal experiments were conducted following the standard operating procedure as outlined by Committee for the Purpose of Supervision and Control Experiments on Animals (CPCSEA), Government of India. The animal experimental protocol was approved by the Institutional Animal Ethics Committee of National Institute of Cholera and Enteric Diseases (Registration No. 68/1999/CPCSEA dated 11-03-1999). Intestines of New Zealand white rabbits weighing about 2 kg were used for mucin preparation. Four to five days old BALB/c mice were used for survival and fluid accumulation studies. For intestine harvesting, the animals were euthanized in a CO_2_-chamber. All efforts were made to minimize suffering during euthanasia.

### Generation of the Δ*chiA2 mutant* strain and its complement

The Δ*chiA2* mutant was constructed as described previously [17]. In brief, an in-frame deletion of 2331 base pair region of *chiA2* gene was created by fusion PCR. The amplicon was cloned into the suicide vector pCVD442 [17]. The resultant chimeric plasmid was transformed into *E. coli* SM10λ*pir* [18] and finally mobilized conjugally to *V. cholerae* N16961. The transconjugants were selected in Ampicillin-Streptomycin double antibiotic Luria Bertani (LB) agar plates. The in-frame deletion of *chiA2* was confirmed by PCR using external primers (Table 1) and nucleotide sequencing. Deactivation of *chiA2* was confirmed by chitinase activity assay. The Δ*chiA2* mutant was complemented with *pchiA2* plasmid to generate a complemented strain following a previously described procedure [17]. Details of the method can be found in File S1.

**Table 1:** List of Primers used in construction of *chiA2* deleted *V. cholerae* strain and cloning *chiA2* in pBAD-TOPO TA expression vector.

Primers	Sequence
chiA2XbaI A F	5 ′-CAGCTCTAGACTCTGATACTGACGTTCGAG-3′
chiA2 B R	5 ′-CCCATCCACTATAAACTAACACAATGGCTTGAGGTGTGAAC-3′
chiA2 C F	5 ′-TGTTAGTTTATAGTGGATGGGGGAACAGGTGGTGATGCTT-3′
chiA2 SACI D	5 ′-GAATCGAGCTCGATTGCATTCCCAATGAG-3′
chiA2 external F	5 ′-GCAGCACCCTCGGCTCC-3′
chiA2 external R	5 ′-CGCGGCACCGAGGTC-3′
chiA2 expression F	5 ′-ATGAATCGAATGACTTTGTGCG-3′
chiA2 expression R	5 ′-TTAATGAGTAGAACAACTCGC-3′

### Purification of wild type ChiA2

*V. cholerae* cells were grown in chitin supplemented alkaline sea-water media (pH 8) with 300 mM salt concentration with shaking at 30°C. The wild type ChiA2 was purified from the 24 h culture supernatant by gel filtration chromatography. The ChiA2 purity was checked by SDS-PAGE and immunoblotting (Fig S1). The fraction with pure ChiA2 was used for further experiments. Details of the method are provided in File S1.

### Purification of rabbit intestinal mucin

Mucin from rabbit intestine was purified by a previously published method [19]. Mucin was extracted from the intestinal scrapings by dissolving it in phosphate buffer saline (PBS) (pH 7.4) containing 6 M guanidine hydrochloride. Iodoacetamide was added and incubated overnight in the dark at room temperature and centrifuged at 45000×*g* for 1 h at 4°C. The supernatant containing crude intestinal mucus was extensively dialyzed first against water and then against PBS to remove the chemicals and free carbohydrate. It was finally lyophilized. The presence of carbohydrate and the protein were determined using the anthrone method and modified Folin-Lowry method respectively. Please see File S1 for details.

### Chitinase activity assay

The N-acetylglucosamine concentration in the reaction mixture and the chitinase activity were determined by previously established Di-nitrosalicylic acid (DNS) method [20]. Chitinase activity was assayed here by estimating reducing sugars by measuring the OD at 540 nm. Varied concentration of purified rabbit [19] and porcine mucin (Sigma, St. Louis, MO) were incubated with 50 µg/ml purified ChiA2 in phosphate buffer pH 7.4 for 3 h at 37°C. The reaction was stopped by adding DNS solution. The mixture was boiled at 100°C for 10 min and cooled by keeping it in ice immediately after boiling. The amount of reducing sugar was then estimated spectrophotometrically. The specific activity of the enzyme was calculated by measuring the amount of GlcNAc produced in µmole/mg of protein/min. The velocity of each reaction was calculated by measuring the amount of GlcNAc produced in µmole/ml of reaction mixture/min. The Michaelis Menten constant K_m_ and the maximum velocity V_max_ were calculated from a Lineweaver Burk plot. The catalytic constant K_cat_ and the catalytic efficiency (K_m_/K_cat_) were also determined. Detailed methods are provided in File S1.

### In-vivo chitinase assay

The *in-vivo* chitinase assay was done by using rabbit intestine. Separated loops were made in the intestine. Each loop was injected with 50 µg/ml ChiA2 in phosphate buffer, pH 7.4. The control loop for each reaction was injected with 50 µg/ml heat inactivated ChiA2 in phosphate buffer pH 7.4. The rabbit intestine was then separated and suspended in a chamber containing warm oxygenated Tyrode-Ringer solution [21] and incubated for 4 h. After every hour one loop was separated and tested for the presence of reducing sugar in the intestinal fluid by the standard DNS method [20].

### Alcian blue staining

Mucin was stained by using alcian blue [22]. Two mg/ml rabbit mucin was treated with 50 µg/ml of ChiA2 in 20 mM phosphate buffer, pH 7.4 at 37°C for 3 h. Ten microliters of ChiA2-treated mucin was blotted on polyvinylidene difluoride (PVDF) membrane (Millipore Co, Bellerica, MA) set on a wet filter paper. The membrane was blocked using 3% BSA for 15 min to reduce the background. The membrane was then stained with 1% alcian blue in 3% acetic acid for 30 min. The membrane was further washed with 3% acetic acid and distilled water, air dried and analyzed by densitometric scanning using Lab works software (UVP).

### HPLC analyses of the end products of ChiA2 treated mucin

HPLC analyses were done to estimate oligosaccharides produced as an end product of ChiA2-mucin reaction. The oligosaccharides were eluted from the column by using a gradient of 60–80% acetonitrile and water. The retention time of each oligosaccharide was compared with commercially available standards. Details of the method are provided in File S1.

### Generation of *V. cholerae* growth curve

Overnight cultures of the wild type *V. cholerae*, Δ*chiA2* mutant and complemented strain were diluted in either M9 minimal media (BD Difco, Sparks, MD) supplemented with mucin or fresh LB broth. The cultures were grown for 72 h at 37°C with shaking. The viable cells were enumerated by plating the cultures at different time points on thiosulfate-citrate-bile-salts-sucrose (TCBS) agar plate. Please see File S1 for details.

### *In vitro* growth assay

The ability of *V. cholerae* to grow in presence of a mucin secreting intestinal cell line was tested. The intestinal epithelial HT29 cells (National Center for Cell science, Pune, India) were cultured in Dulbecco's Modified Eagle's Medium (DMEM), supplemented with 10% fetal bovine serum (FBS) (HiMedia, Mumbai, India), 1% non-essential amino acid and 1% Penicillin/Streptomycin mixture at 37°C under 5% CO_2_ in a humidified CO_2_ incubator. The HT29 cells were cultured up to 80% confluency. Before the experiment, the cells were kept in DMEM containing 0.5% FBS for 18 h starvation. Serum-starved HT29 cells were then incubated with different dilutions (1×10^5^, 1×10^6^, 1×10^7^ and 1×10^8^ CFU/ml) of *V. cholerae*, Δ*chiA2* mutant or complemented strain for 12 h. After 12 h the culture supernatants containing the unbound bacteria were collected and culture plates were incubated with 0.1% Triton X-100 [23] for 2–3 min, vortexed mildly and the contents of the wells containing the bound bacteria were collected. Collectively the unbound and bound bacteria were washed with phosphate buffered saline, diluted (10^−5^) and plated on TCBS agar plate to enumerate the total viable *V. cholerae*. Untreated HT-29 cell culture media was used as a negative control.

### *In vivo* assay using infant mice

Mouse infection and bacteria recovery were performed as described previously [24]. In brief, 4 to 5 days old BALB/c mice were separated from their mothers 2 h before oral inoculation with 100 µl of sterile PBS containing 10^6^ CFU/ml of *V. cholerae*, Δ*chiA2* mutant or the complemented strain. Infected mice were kept in the absence of their mothers and sacrificed after 16 h post infection. Their entire intestines were removed and homogenized in 2 ml LB containing 20% glycerol without removing the intestinal contents. Serial dilutions were plated onto TCBS agar to enumerate the viable bound and unbound *V. cholerae* CFU/ml [24]. Mice inoculated with sterile PBS were used as negative controls.

Fluid accumulation assay was done in infant mice as described previously [25]. Mice were sacrificed 4, 8, 12 and 18 h post infection, weighed and their entire intestines were removed. Each of the separated intestines was weighed. Fluid accumulation was calculated as intestinal weight/(whole body weight)-(intestinal weight) [25]. Mice inoculated with sterile PBS were used as negative controls.

### Rabbit ileal loop assay

The rabbit ileal loop assay was performed in young New Zealand white rabbits (2.5 kg) by a method described previously [26]. *V. cholerae* and Δ*chiA2* mutant cultures were grown over night in LB broth. The overnight cultures were centrifuged and washed with PBS. The washed *V. cholerae* and Δ*chiA2* mutant cells were inoculated in a rabbit ileum in a concentration of 1×10^9^ CFU/ml. PBS-inoculated loop was used as a negative control. The animal was sacrificed after 18 h and the enterotoxic response was determined by measuring the fluid accumulation (FA) ratio. The FA ratio was calculated by the following formula: FA/cm  =  volume of fluid accumulation (ml)/intestinal length (cm) [27]. A ratio of greater than 1.0 indicated a strong positive response, while a negative response was defined as FA ratio of less than 0.2.

### Histological studies

Tissue samples (2 cm in length) from rabbit ileal loop assays were collected and placed in 10% neutral-buffered formalin for histological analysis. Tissues were embedded in paraffin and processed following the standard protocol. Sections of 3 to 4 µm thick were prepared using a Leica rotary microtome were stained with hematoxylin and eosin and examined under a light microscope. Photographs were taken under different magnifications with a Leica DMLB microscope (Solms, Germany), equipped with a digital imaging system.

### Statistical analysis

All the experiments were repeated for at least three times. Data are expressed as means ± standard error (SE). One way ANOVA was used to analyze the data wherever applicable. A *P* value of <0.05 was considered statistically significant.

## Results

### ChiA2 utilizes mucin as a substrate

**Figure 1:**
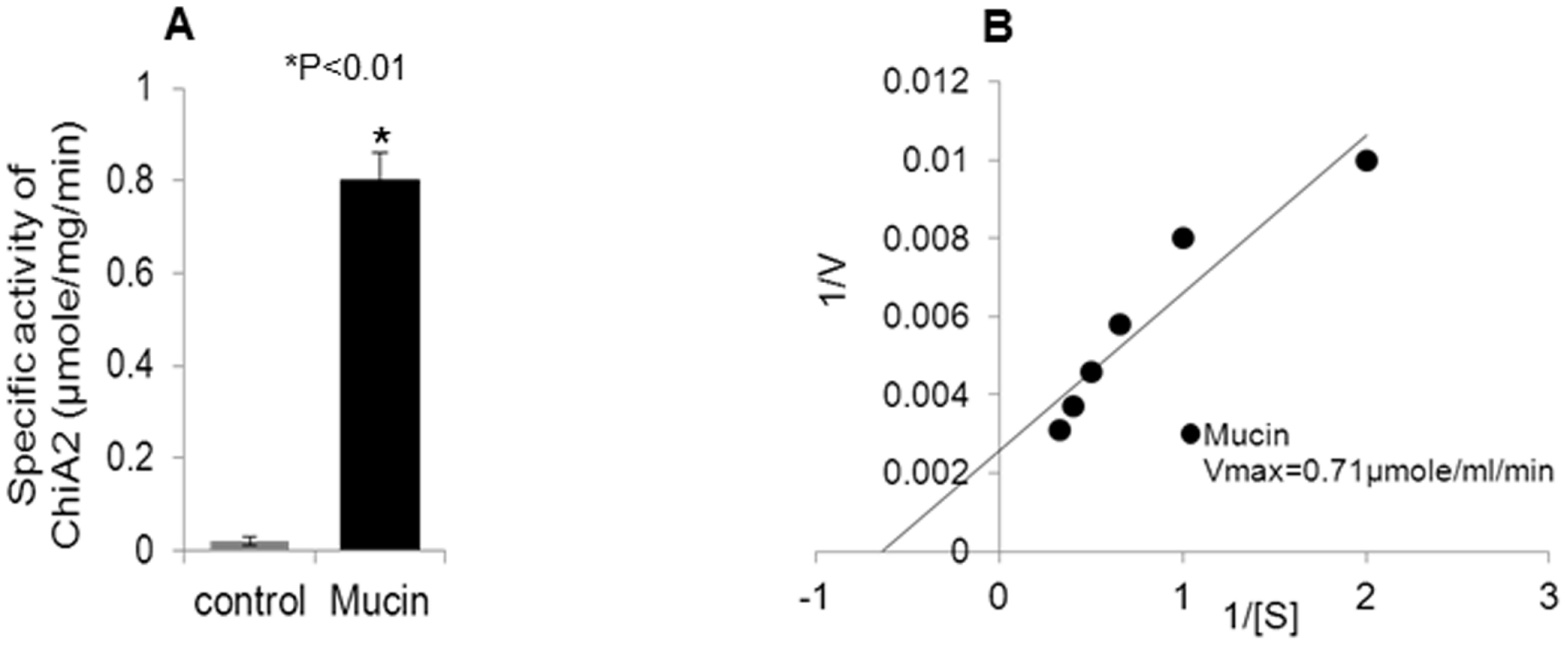
ChiA2 can use mucin as a substrate. **A**. This diagram shows the specific activity of ChiA2 when mucin was used as substrate. In the control experiment heat inactivated ChiA2 was used instead of active ChiA2. The assay was done as described in materials and methods. The experiment was repeated thrice (n = 3) and the data expressed as means ± SEM. **B**. This figure shows the Lineweaver Burk plot of ChiA2-mucin reaction. The plot was generated by plotting inverse of different substrate (mucin) concentrations (0.5 mg/ml, 1 mg/ml, 1.5 mg/ml, 2 mg/ml, 2.5 mg/ml and 3 mg/ml) in the X-axis and inverse of each reaction velocity in Y axis.

The degradation of carbohydrate moiety of mucin by ChiA2 was assayed colorimetrically using DNS solution. The result showed presence of reducing sugars in the end product mixture that indicated ChiA2 was active against purified mucin. The specific activity of ChiA2 against mucin was 0.79 µmole/mg/min. The negative control, treated with the heat inactivated enzyme, did not show the presence of reducing sugars (Fig. 1A) indicating that the release of reducing sugars from mucin was induced by ChiA2. The Michaelis-Menten constant, K_m_ and the maximum velocity V_max_ for the ChiA2-mucin reaction was determined by the Linewever Burk plot with varying mucin concentrations (Fig. 1B). The K_m_ was 3.0±0.5 mg and the V_max_ of the reaction was 0.71 µmole/ml/min and also the catalytic constant K_cat_ and the catalytic efficiency (K_cat_/K_m_) determined was (1.4±0.2) ×10^−2^/sec and 4.6×10^−3^/sec/mg respectively.

### ChiA2 possesses mucin deglycosylase activity

**Figure 2:**
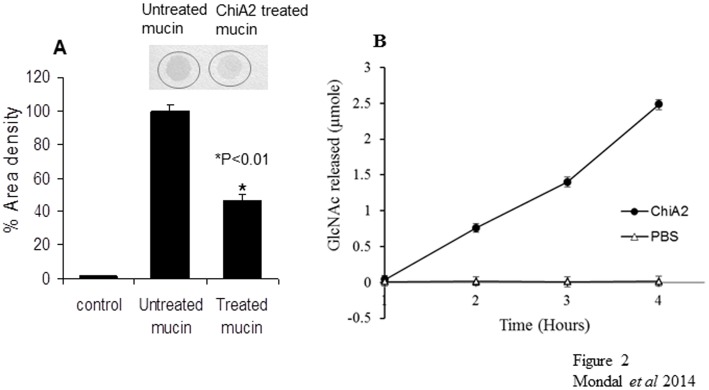
Mucin deglycosylase activity of ChiA2. **A**. This shows the graphical representation of % area density of the alcian blue stained spots (the above picture) of control, ChiA2-untreated and treated rabbit mucin respectively (from left). A 50-fold decrease in the % area density of the spot was observed for ChiA2-treated rabbit mucin compared to the untreated one. The mucin treatment was done as mentioned in materials and methods section. The % area density for each spot was calculated using Lab works software after alcian blue staining. **B**. This is the graphical representation of the amount of GlcNAc released by ChiA2 from in-vivo rabbit intestinal mucin (•) with respect to the control (Δ) with increasing incubation time. The graph shows a significant increase in reducing sugar concentration in the reaction loop containing active ChiA2 compared to the control loop containing the heat deactivated enzyme in phosphate buffer. The assay was done as mentioned in materials and methods. Each of the experiment was repeated thrice (n = 3) and the data expressed as means ± SEM.

When ChiA2 treated mucin was stained with alcian blue, it was found to uptake the stain very poorly relative to the untreated mucin. Densitometric analysis of the stained spots using Labworks 4.6 software showed 65% decrease in staining density of ChiA2 treated mucin compared to the untreated one (Fig. 2A). Alcian blue is a synthetic basic dye [28]. At pH 2.5 (3% acetic acid) alcian blue stains both sulfated and carboxylated acid mucopolysaccharides [29]. The histochemical reactivity of the mucins with alcian blue depends largely upon the carbohydrate composition of the mucin [30]. So it is possible that, if ChiA2 disrupts the outer most carbohydrate covering of mucin, it will fail to uptake the stain.

In vivo assay for mucin deglycosylase activity of ChiA2 was carried out in rabbit intestine. ChiA2 did not show any significant activity up to 1 h of incubation. After 1 h, an increase in reducing sugar concentration was observed in the reaction mixture inside the intestine. The control loop containing the heat deactivated enzyme in phosphate buffer did not produce any reducing sugar even after 4 h of incubation (Fig. 2B).

### ChiA2 releases GlcNAc, (GlcNAc)_2_ and (GlcNAc)_3_ from mucin

**Figure 3:**
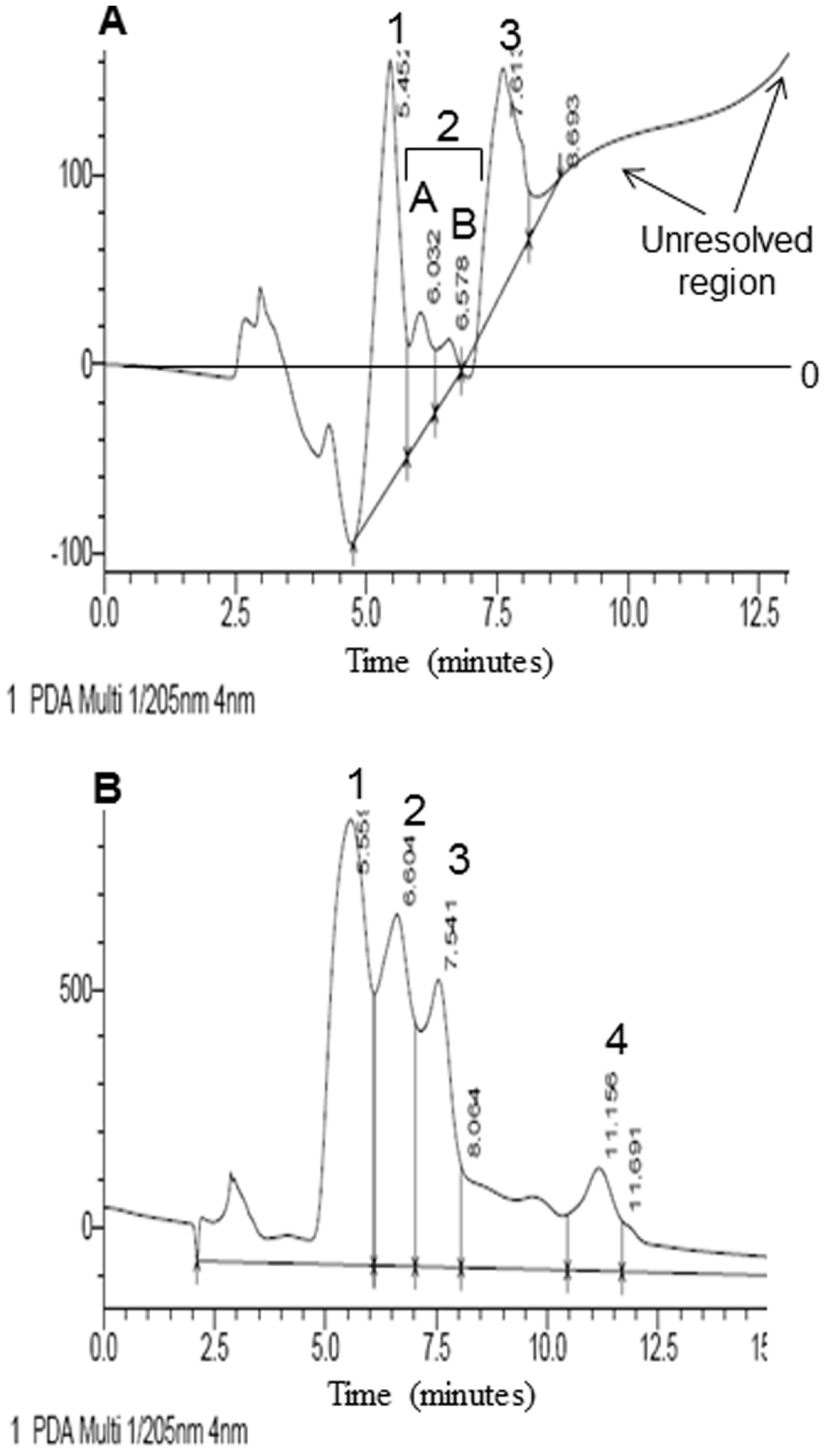
HPLC analysis of end products of ChiA2-treated mucin. The mucin-ChiA2 Reactions were carried as mentioned in the materials and methods section. The end products were analyzed using a 4.6 nm×250, 5 µm Zorbax carbohydrate analysis column (Agilent Technologies, Waldbrown, Germany) connected to a Shimadzu Prominence 20A; HPLC system. **A**. This figure shows the HPLC chromatogram of the end products of ChiA2 treated mucin. The chromatogram shows four distinct peaks and an unresolved region. Peak 1 with retention time 5.4 minutes indicates presence of GlcNAc. Peak 2 indicates presence of (GlcNAc)2 which is divided into two separate peaks: peak 2A and peak 2B. Peak 2A with retention time 6.0 minutes probably is for the β enantiomer and peak 2B with retention time 6.57 minutes is for α enantiomer of (GlcNAc)_2_. Peak 3 with retention time 7.6 minutes indicates the presence of (GlcNAc)_3_. **B**. This is the HPLC chromatogram of standards of GlcNAc, (GlcNAc)_2_, (GlcNAc)_3_ and (GlcNAc)_6_. The chromatogram shows 4 distinct peaks. Peak 1 with retention time 5.5 minutes is for GlcNAc. Peak 2 with retention time 6.6 minutes is for α-(GlcNAc)_2_. Peak 3 with retention time 7.5 minutes is for (GlcNAc)_3_ and peak 4 with retention time 11.1 minutes is for (GlcNAc)_6_.

The end-products of ChiA2-mucin reaction were analyzed by HPLC and the components identified by corresponding standards (Fig 3A). Peak 1 with retention time 5.4 min corresponded to GlcNAc standard (Fig 3B). GlcNAc was the abundant component and occupied approximately 40% of the total peak area. Peak 2A and 2B eluted in close proximity with the retention time of 6.0 and 6.57 min. Peak 2B with retention time 6.57 min corresponded to α-(GlcNAc)_2_ (Fig 3A) (Table 2). Peak 2A eluted at 6.03 min; in absence of a standard and in view of the close proximity to peak 2B we inferred that it corresponded in all probability to β-(GlcNAc)_2_. The amount of (GlcNAc)_2_ was found to be very low compared to GlcNAc and (GlcNAc)_3_. The peak occupied only 4.9% of the total peak area which was significantly less compared to the other two peaks (Table 2). Peak 3 with a retention time of 7.6 min corresponded to chitotriose (GlcNAc)_3_ which occupied 30.8% of the total peak area (Fig 3A) (Table 2). The presence of an unresolved region was observed after peak 3 (Fig 3A) which might be due the presence of other large chain N-linked or O-linked sugar moieties which could not be separated by the column.

**Table 2:** HPLC analysis of the end products of ChiA2 treated mucin.

			Standards		ChiA2-mucin reaction	
*Peak number*	*Sub peak*	*Peak Identity*	*Ret. Time (mins)*	*% Area*	*Ret. Time (mins)*	*% Area*
**1.**		(GlcNAc)	5.5±0.05	35.8	5.45±0.05	40.1
**2.**	a	(GlcNAc)_2_α	NO*	19.7	6.03±0.03	4.9
	b	(GlcNAc)_2_β	6.6±0.03		6.57±0.03	
**3.**		(GlcNAc)_3_	7.5±0.02	16.1	7.61±0.02	30.8
**4.**		(GlcNAc)_6_	11.15±0.03	6.5	NO*	-

Reactions were carried out in 20 mM phosphate buffer pH 7.4 with 1 mg/ml rabbit mucin as substrate and incubated at 37°C with 50 µg/ml of pure ChiA2 for 3 hours. The end products were analyzed using a 4.6 nm×250, 5 µm Zorbax carbohydrate analysis column (Agilent Technologies, Waldbrown, Germany) connected to a Shimadzu Prominence 20A; HPLC system. The reaction was stopped by 10% TCA loaded into the HPLC column and eluted using a gradient of 60–80% acetonitrile in water as mobile phase. Each experiment was conducted maintaining same conditions and repeated 3 times.

*Not Observed.

### *V. cholerae* utilizes mucin as nutrient source

**Figure 4:**
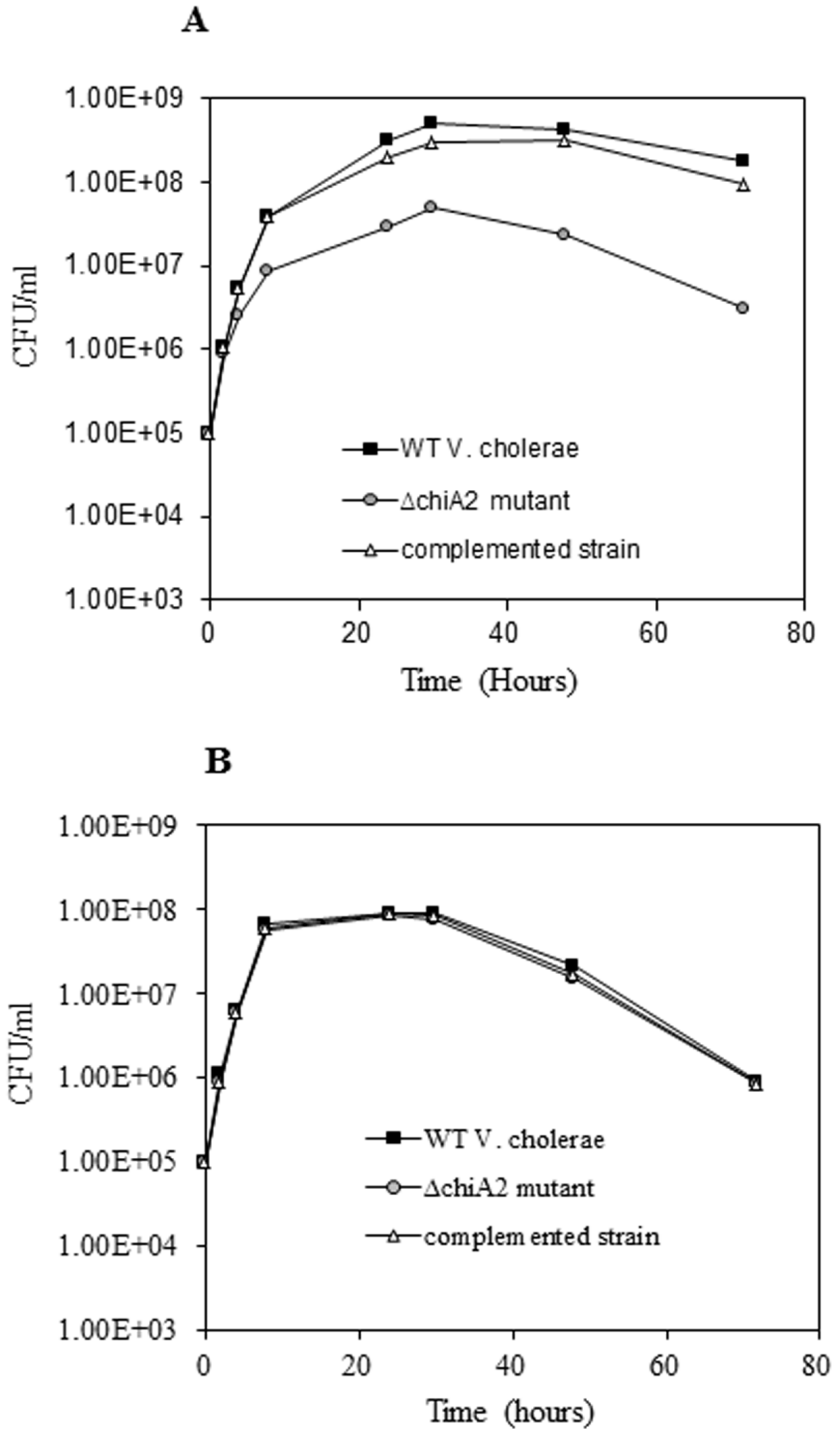
Utilization of mucin as nutrient for growth by *V. cholerae*. *V. cholerae* wild type, Δ*chiA2* mutant and the complemented strain were separately grown in LB upto log phase. The log phase cultures of the wild type *V. cholerae*, Δ*chiA2* mutant and the complemented strain were centrifuged and washed in PBS. The minimal media supplemented with 2% (w/v) porcine mucin and fresh LB broth were inoculated separately with equal number of washed *V. cholerae* wild type and Δ*chiA2* mutant and complemented strain. The number of bacteria survived with increasing incubation time was detected by plate count method for all of the strains. The viable counts of bacteria were graphically represented. **A**. Viable count when mucin used as nutrient. **B**. Viable count when grown in LB broth. Each of the experiment was repeated thrice (n = 3) and the data expressed as means ± SEM.

The Δ*chiA2* mutant grew very poorly in minimal media supplemented with mucin compared to the wild type strain. At 72 h, the total viable count of wild type *V. cholerae* was 1.8×10^8^ CFU/ml, whereas it was only 3×10^6^ CFU/ml for the Δ*chiA2* mutant, showing a 60-fold difference in growth (P<0.01) (Fig. 4A). In contrast, a wild type *V. cholerae* and its Δ*chiA2* mutant had similar growth patterns in LB broth (Fig. 4B) suggesting that *V. cholerae* could utilize mucin as a nutrient and its utilization involved ChiA2.

### ChiA2 plays a role in *V. cholerae* intestinal survival

**Figure 5:**
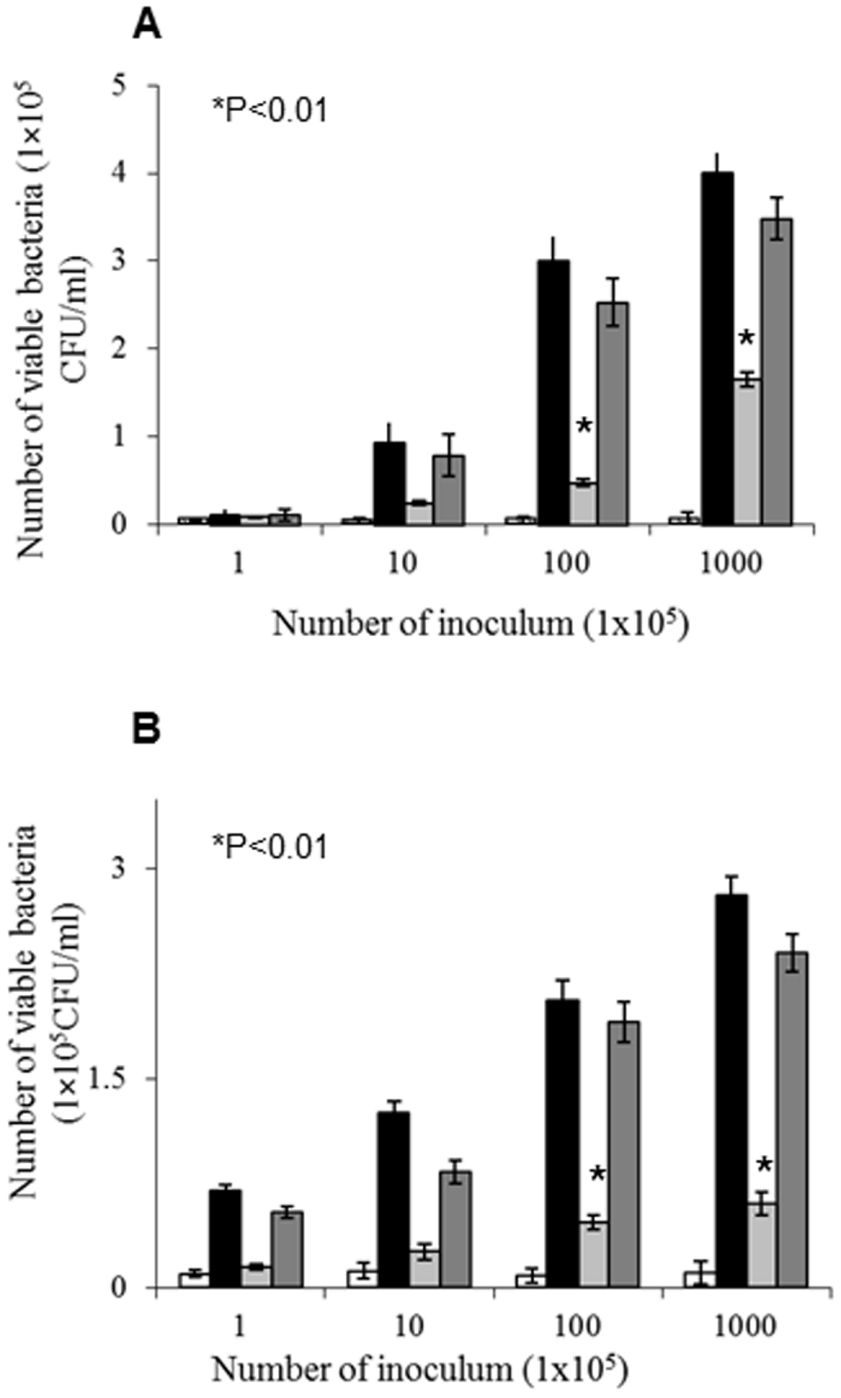
Role of ChiA2 in *V. cholerae in vitro* and *in vivo* proliferation. **A**. Comparative study of the survival of wild type and Δ*chiA2* mutant in human intestinal HT29 cell line. The graph represents a dose dependent survival of wild type *V. cholerae* and Δ*chiA2* mutant and also a complemented strain. **B**. Comparative study of dose dependent mice intestinal survival of wild type *V. cholerae*, Δ*chiA2* mutantand the complemented strain. For the experiment, 5 days old suckling mice were fed with different dilutions of the mentioned strains and sacrificed after 16 hours. The viability of the strains was measured by cell count method. Bars: (□) PBS; (▪) wild type *V. cholerae*; (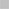 ) Δ*chiA2* mutant (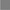) complemented strain. Each of the experiment was repeated thrice (n = 3) and the data expressed as means ± SEM.

Growth phenotype study of *V. cholerae* in mucin supplemented minimal media suggested that *V. cholerae* utilized mucin as a nutrient source. When *V. cholerae* was incubated for 12 h with HT29 cells, a human intestinal cell line specializing in synthesis and secretion of mucin, the multiplication of the wild type cells was 5.8-fold more than that of the Δ*chiA2* mutant when incubated with 1×10^7^ bacteria (P<0.01). However, the complemented strain grew normally indicating reversal of the phenotype (Fig 5A).

Similar results were obtained when intestine of the mice was infected with *V. cholerae* and Δ*chiA2* mutant. Up to 6-fold fewer bacteria were recovered from cells infected with the Δ*chiA2* mutant compared to the wild type when infected with 1×107 bacteria (p<0.01). Infection with complemented strain showed significant reversal of the phenotype with a 5-fold increase in recovery with respect to the Δ*chiA2* mutant (Fig 5B). This result indicates that ChiA2 is important for *V. cholerae* colonization in the mice intestine.

### *V. cholerae* shows reduced pathogenesis in absence of *chiA2*

**Figure 6:**
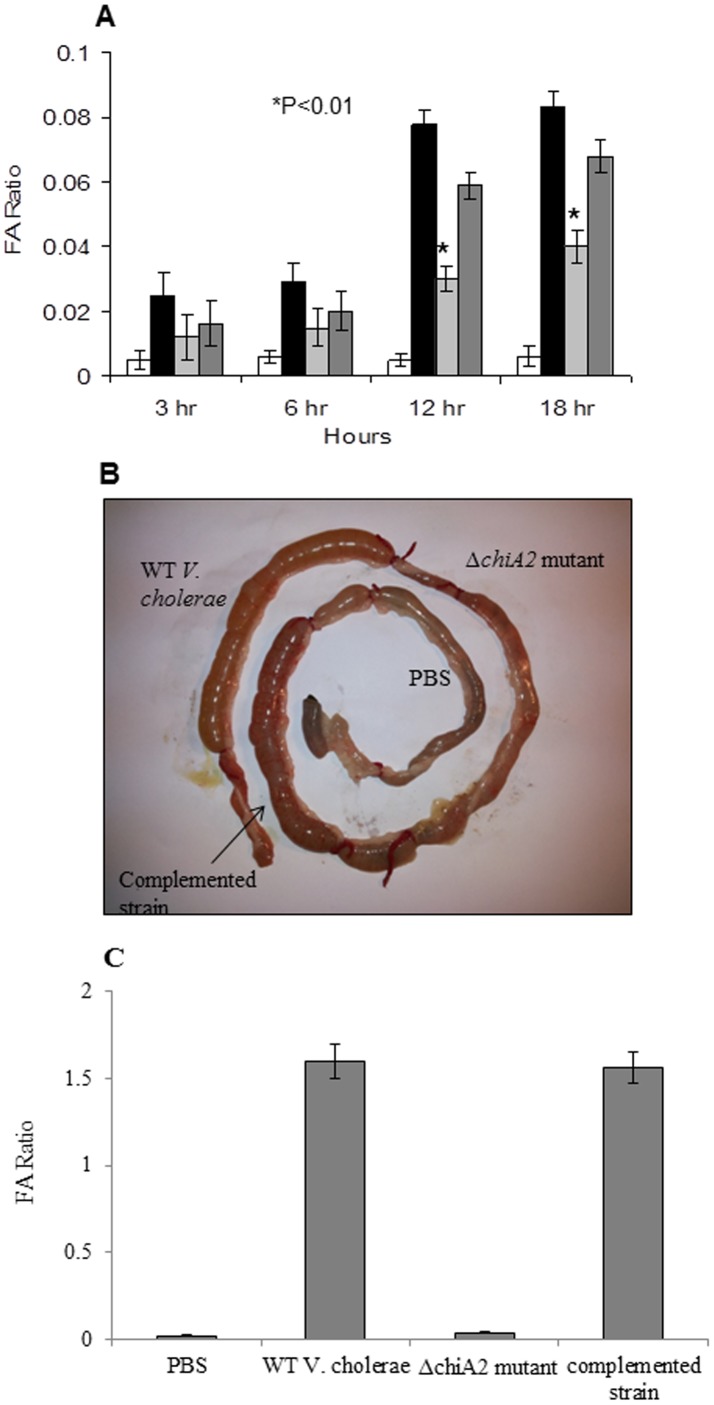
Fluid accumulation studies. **A**. This is the graphical representation of fluid accumulation ratio in infant mice model. For fluid accumulation studies 5 days old infant mice were infected with 100 µl of 1×10^6^ CFU/ml of wild type *V. cholerae*, Δ*chiA2* mutant, and the complemented strain. Mice were sacrificed after 3, 6, 12 and 18 hours. The fluid accumulation calculated and presented graphically. In all the above three experiments PBS was used as negative control and the data expressed as means ± SEM of three independent experiments (P<0.01). **B**. Rabbit ileal loop response of wild type *V. cholerae* cells (1×10^9^ CFU/ml) showing hemorrhagic fluid accumulation whereas, infection with Δ*chiA2* mutant (1×10^9^ CFU/ml) shows significant decrease in fluid accumulation. Infection with the complemented strain showed similar hemorrhagic fluid accumulation like the wild type strain. PBS was used as negative control. The experiment was repeated thrice (n = 3). **C**. This is the graphical representation of fluid accumulation ratio in rabbit ileal loop. The fluid accumulation ratio for the Δ*chiA2* mutant (FA/cm ratio: 0.04±0.01) was 40-fold less compared to the wild type *V. cholerae* (FA/cm ratio: 1.6±0.2). The fluid accumulation ratio for the complemented strain was similar with the wild type. PBS was used as negative control (FA/cm ratio: 0.02±0.008). The experiment was repeated thrice (n = 3) and the data expressed as means ± SEM.

Pathogenesis was studied based on fluid accumulation in infant mouse model. Fluid accumulation with Δ*chiA2* mutant was 40-fold less compared to the wild type *V. cholerae* infection at 12 h post infection. Fluid accumulation was 50-fold less for Δ*chiA2* mutant with respect to wild type infection at 18 h post infection. Infection with the complemented strain showed significant reversal of the phenotype at both the time points (Fig 6A).

Pathogenesis was further confirmed by rabbit ileal loop model. Infection with wild type *V. cholerae* showed hemorrhagic fluid accumulation after 18 h of infection (Fig 6B). Infection with the equal number of Δ*chiA2* mutant with equal incubation time showed 40-fold decrease in fluid accumulation (Fig. 6C). Infection with the complemented strain showed significant reversal of the phenotype.

### Infection with Δ*chiA2* mutant caused less damage to the rabbit ileal tissue compared to the wild type strain

**Figure 7:**
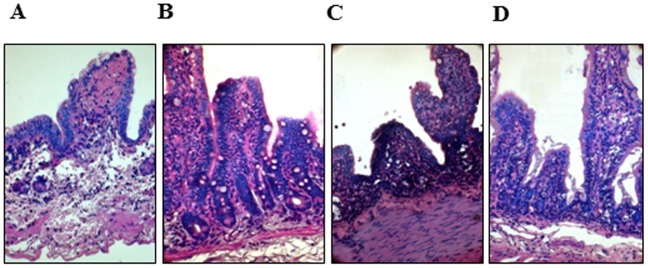
Histological study of rabbit ileal tissues. Panels show photomicrographs of histology of rabbit ileal loop tissue after infection with **A**. wild type *V. cholerae* showing hemorrhagic fluid accumulation (Fig. 5B). Extensive damage of mucosa with damage to the villus structure and gross dilatation of villi with hemorrhage and necrotic changes in villus, lamina, propria, mucosa and sub-mucosal area was observed. **B**. Almost normal villous and mucosal structure was observed in ileal tissues infected with Δ*chiA2* mutant though some congestion and scattered hemorrhagic change was noticed in villus, lamina, propria and rest of the mucosa. **C**. complemented strain showing hemorrhagic fluid accumulation. Extensive damage of mucosa with damage to the villus structure and gross dilatation of villi with hemorrhage and necrotic changes in villus, lamina, propria, mucosa and sub-mucosal area was observed. **D**. PBS treated ileal tissue showed normal gut mucosa.

Histopathological analysis of the rabbit ileum revealed that the wild type *V. cholerae* caused extensive damage to all the layers of the mucosa. A gross dilatation of villi with hemorrhage and necrotic changes in villus, lamina, propria, mucosa and sub-mucosal area were observed (Fig 7A). On the other hand, analysis of ileal tissues treated with Δ*chiA2* mutant showed almost normal villus and mucosal structure though minor congestion and scattered hemorrhagic change was noticed in villus, lamina, propria and rest of the mucosa (Fig 7B). PBS treated ileal tissue did not show any alteration of the gut mucosa (Fig 7C).

## Discussion

The extracellular chitinases ChiA1 and ChiA2 are essential for *V. cholerae* environmental survival [2]. However, their role in host infection and pathogenesis remains unclear. We used ChiA2 as a representative chitinase for this study since it is maximally expressed in the rabbit intestine compared to other chitinases [31]. We have observed ChiA2 is active against mucin *in vitro* and *in vivo* conditions. Previous studies from other laboratories have speculated that inside the host chitinases and CBP of pathogenic bacteria interact with the carbohydrate moiety of glycoproteins bearing chemical resemblance to chitin [10], [12]. Our results indicate that ChiA2 hydrolyzed the β-1, 4- linkages connecting GlcNAc moieties present in mucin. Mucolytic activity of bacterial chitinases was reported previously but only in assistance of proteases [32]. However ChiA2 did not require any proteolytic help in mucin deglycosylation.

ChiA2 released significant amounts of GlcNAc and (GlcNAc)_3_ from mucin. These were similar to the end products of chitin hydrolysis by ChiA2 (Fig S2). However, the end products of ChiA2 treated chitin and mucin could not be compared qualitatively and quantitatively since the composition of chitin and mucin are different. Chitin is a homo-polymer of GlcNAc residues only, whereas the oligosaccharide moieties of mucin contains both N-linked and O-linked GlcNAc residues connected through β-1, 4 and β-1, 3 linkages [33], [34]. ChiA2 activity on mucin probably releases the N-linked or O-linked sugar moieties along with GlcNAc and its short chain oligomers like (GlcNAc)_2_ and (GlcNAc)_3_. Removal of these sugar moieties might eventually alter the outer carbohydrate layer of mucin resulting in poor uptake of the alcian blue stain. It was reported earlier that the histochemical activity of mucin with Alcian blue depended largely upon the carbohydrate composition of the mucin [30].

Our results showed that *V. cholerae* could actively utilize mucin as a nutrient and in absence of *chiA2* poor growth was evidenced in mucin containing media. The use of mucin as a nutrient by intestinal microbes was described previously where mucin served as the endogenous carbon and energy source [35], [36], [37]. It was also reported that *Clostridium perfringens*, an opportunistic intestinal pathogen was able to grow on medium with mucin as a substrate [35]. However, utilization of mucin as a nutrient source by *V. cholerae* was not demonstrated before. Probably, ChiA2 hydrolyzed oligosaccharide moieties of mucin releasing GlcNAc which could be readily utilized by *V. cholerae* as a nutrient and this supported *V. cholerae* growth in media supplemented with mucin. The N-acetylglucosamine rich side chains of mucin can act as a source for nitrogen and carbon [38], [39]. For some pathogens, GlcNAc has been reported to be utilized more efficiently than glucose [36]. *V. cholerae* has an efficient system for GlcNAc catabolism which includes functional involvement of various enzymes encoded by *nag* genes (*nag* A, B, C, D, E) [40]. The *nag* genes and also the chitinase genes including *chiA2* are up-regulated in presence of GlcNAc [2]. A recent study has demonstrated that a two gene-cluster co-ordinated this catabolic pathway [41] So, it is possible that ChiA2 is indirectly involved in GlcNAc utilization pathway by releasing GlcNAc from mucin and therefore supporting the growth and survival of *V. cholerae* in mucin containing media.

Our results indicate that *V. cholerae* might survive in the intestine using mucin as a nutrient source. We observed that the Δ*chiA2* mutant showed slower proliferation and survived poorly both in mice intestine and human intestinal HT29 cells. There are reports that chitinases enhanced the survival and growth of pathogens in mammalian host cells [12], [42], but role of the enzyme in pathogenesis is only speculated. The role of a chitinase in this aspect is described here for the first time.

Apparently it seems that the deletion of *chiA2* has made the mutant either weak or binding-deficient. However, we found that Δ*chiA2* mutant grew well in enriched medium (LB broth) and produced similar amount of cholera toxin (Fig S3). Further, *V. cholerae* and the Δ*chiA2* mutant did not show any difference in initial adherence (Fig S4). Hence, we predict that in absence of ChiA2, these bacteria are unable to utilize mucin and the mutant does not grow well in the intestinal niche. As a result, the Δ*chiA2* mutants were recovered in fewer numbers and appear less pathogenic in comparison to the wild type strains.

Intestinal fluid accumulation study in infant mice along with adult rabbits indicates that Δ*chiA2* mutant is less virulent than the wild type strain. Role of bacterial chitinases in host pathogenesis is recently reported but the mechanisms are not clear. Whether a common mechanism exists for pathogenesis or several organism specific mechanisms play the role are yet to be revealed. A study has reported about the involvement of chitinases in bacterial virulence and pathogenesis through modulation of host innate immunity [11] whereas, other studies have indicated bacterial chitinases are mainly required for survival, persistence and growth in the host [12], [42]. Yet another study pointed *E. coli* chitinase ChiA to play a role as virulence-factor and promote bacterial adhesion to mucosal tissues [8]. However, further studies are needed to understand the exact mechanisms of how ChiA2 of *V. cholerae* contribute in host infection.

*V. cholerae* chitinases are commonly viewed as enzymes needed for chitin hydrolysis and environmental survival. Our study here describes an additional role of *V. cholerae* extracellular chitinase ChiA2 in intestinal survival and virulence. ChiA2 can degrade the oligosaccharide moieties of mucin releasing GlcNAc which is possibly utilized as a nutrient by *V. cholerae*. In addition, activity of ChiA2 may make the host intestinal mucin layer more susceptible possibly by decreasing the electroviscous property of mucus, helping in *V. cholerae* virulence. Understanding the role of *V. cholerae* chitinases in pathogenesis will open new avenues of therapeutic intervention during cholera.

## Supporting Information

Figure S1 **SDS-PAGE and Western Blot of pure ChiA2 (wild type).** The purity of ChiA2 was checked by SDS-PAGE and the presence of ChiA2 was confirmed by Western blot.
(TIF)

Figure S2 **HPLC chromatogram of the end products of chitin after ChiA2 treatment.** This figure shows the HPLC chromatogram of the end products of ChiA2 treated chitin. The chromatogram shows 4 distinct peaks. Peak 1 with retention time 5.5 minutes indicates presence of GlcNAc. Peak 2 indicates presence of (GlcNAc)_2_ which is divided into two separate peaks: peak 2a and peak 2b. Peak 2a with retention time 6.2 minutes probably is for the β enantiomer and peak 2b with retention time 6.57 minutes is for α enantiomer of (GlcNAc)_2_. Peak 3 with retention time 7.42 minutes indicates the presence of (GlcNAc)_3_.
(TIF)

Figure S3 **Comparison of cholera toxin gene expression in wild type** ***V. cholerae*** **and Δ** ***chiA2*** **mutant.** This figure shows comparative graphical representation of *ctx* gene expression in wild type *V. cholerae*, Δ*chiA2* mutant and the complemented strain. The *ctx* expression was measured quantitatively by real time PCR using Power SYBR Green PCR master Mix in a 7500 real time PCR detection system.
(TIF)

Figure S4 **Initial adherence of** ***V. cholerae*** **on HT29 cells.** This figure shows the graphical representation of the comparative study of initial adherence of wild type *V. cholerae*, Δ*chiA2* mutant and the complemented strain on HT29 human intestinal epithelial cells. The bound bacteria were enumerated by plate count method using TCBS agar plates.
(TIF)

File S1 **Elaborated Methodology.**
(DOC)
