# Supplementary figures and images for: The Vibrio cholerae Extracellular Chitinase ChiA2 Is Important for Survival and Pathogenesis in the Host Intestine
Source: PLoS One. 2014 Sep 22;9(9):e103119. doi: 10.1371/journal.pone.0103119 (PMC4170974; doi:10.1371/journal.pone.0103119)

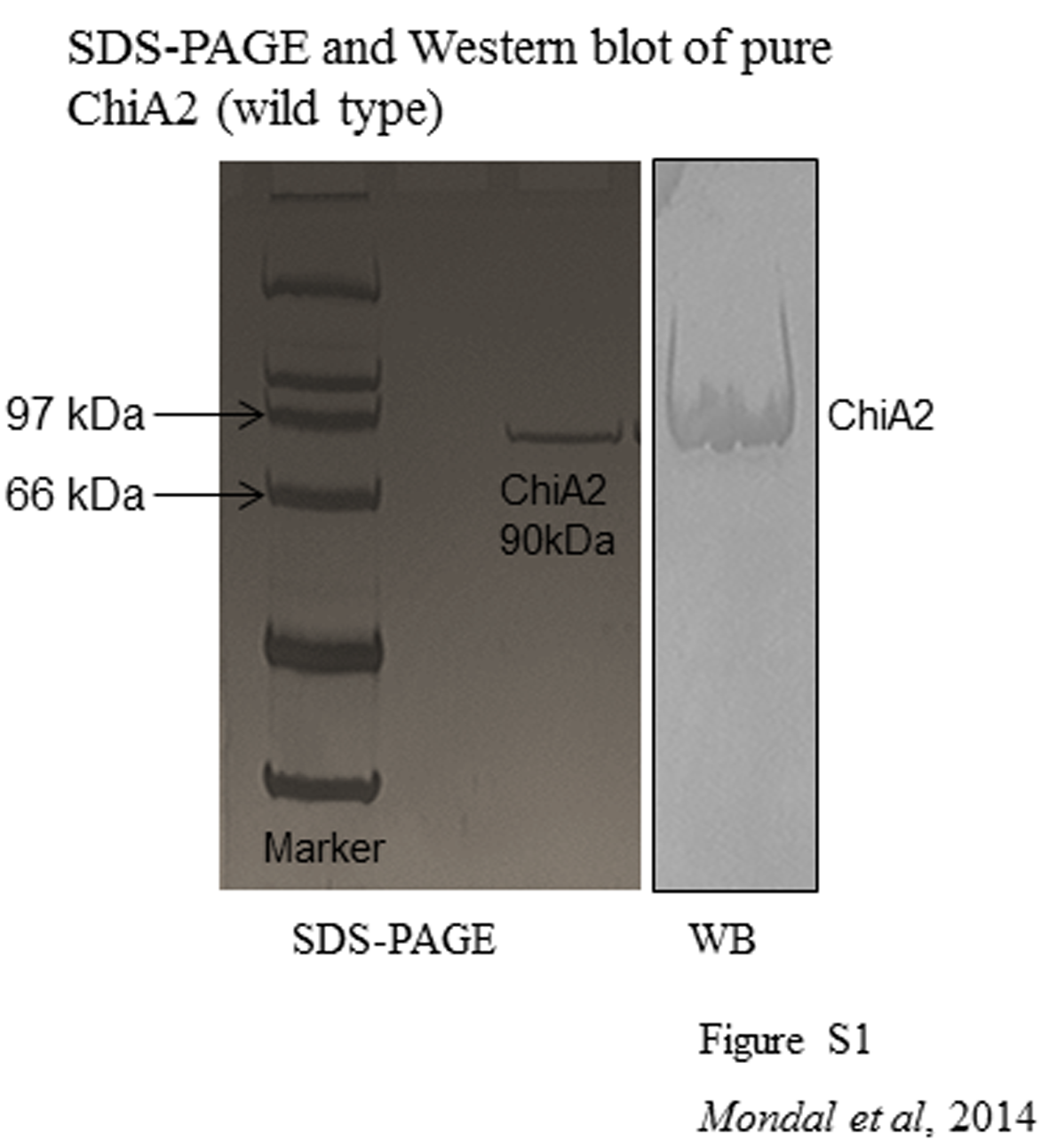

Supplement: Figure S1 — SDS-PAGE and Western Blot of pure ChiA2 (wild type). The purity of ChiA2 was checked by SDS-PAGE and the presence of ChiA2 was confirmed by Western blot. (TIF) [file pone.0103119.s001.tif]

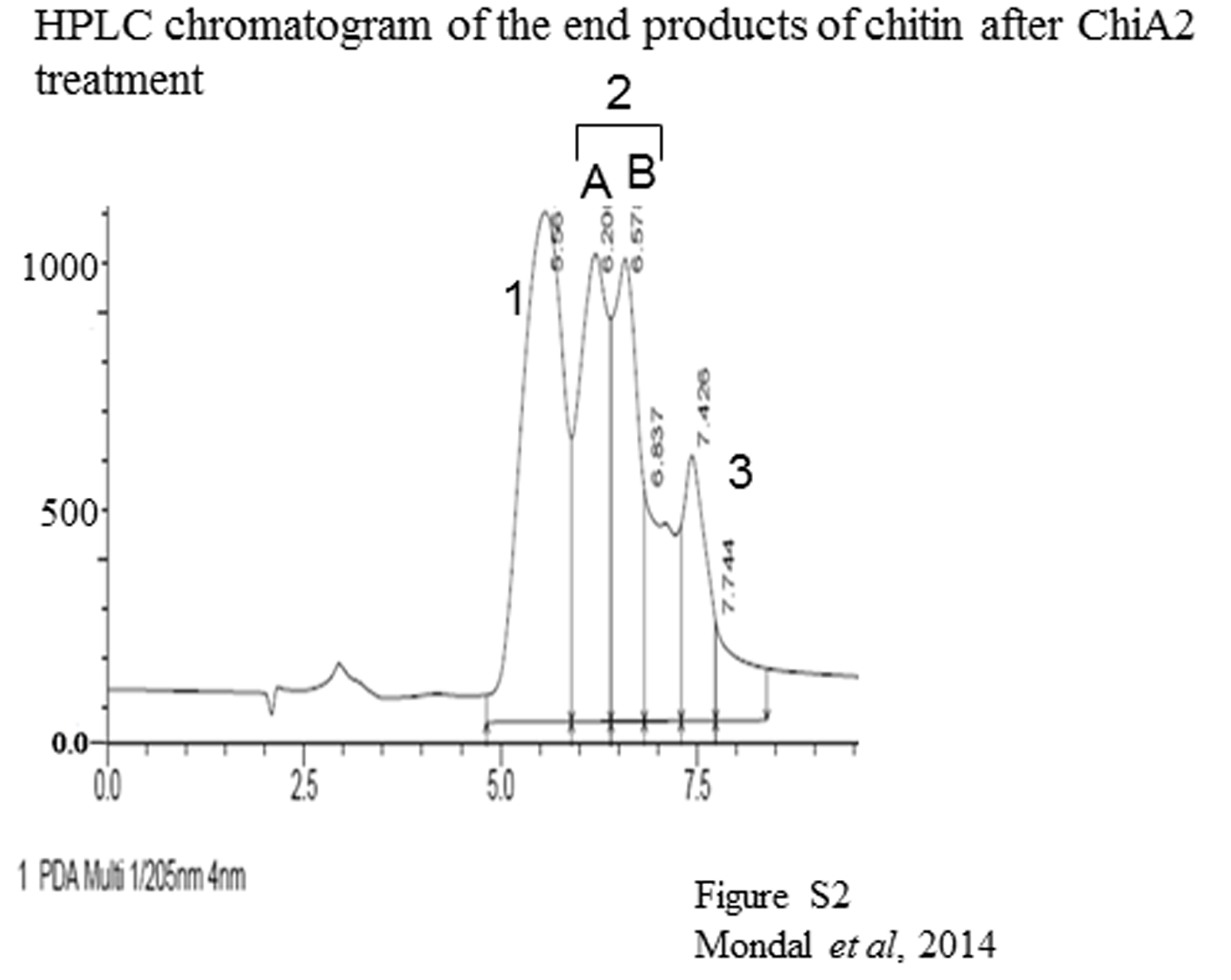

Supplement: Figure S2 — HPLC chromatogram of the end products of chitin after ChiA2 treatment. This figure shows the HPLC chromatogram of the end products of ChiA2 treated chitin. The chromatogram shows 4 distinct peaks. Peak 1 with retention time 5.5 minutes indicates presence of GlcNAc. Peak 2 indicates presence of (GlcNAc)2 which is divided into two separate peaks: peak 2a and peak 2b. Peak 2a with retention time 6.2 minutes probably is for the β enantiomer and peak 2b with retention time 6.57 minutes is for α enantiomer of (GlcNAc)2. Peak 3 with retention time 7.42 minutes indicates the presence of (GlcNAc)3. (TIF) [file pone.0103119.s002.tif]

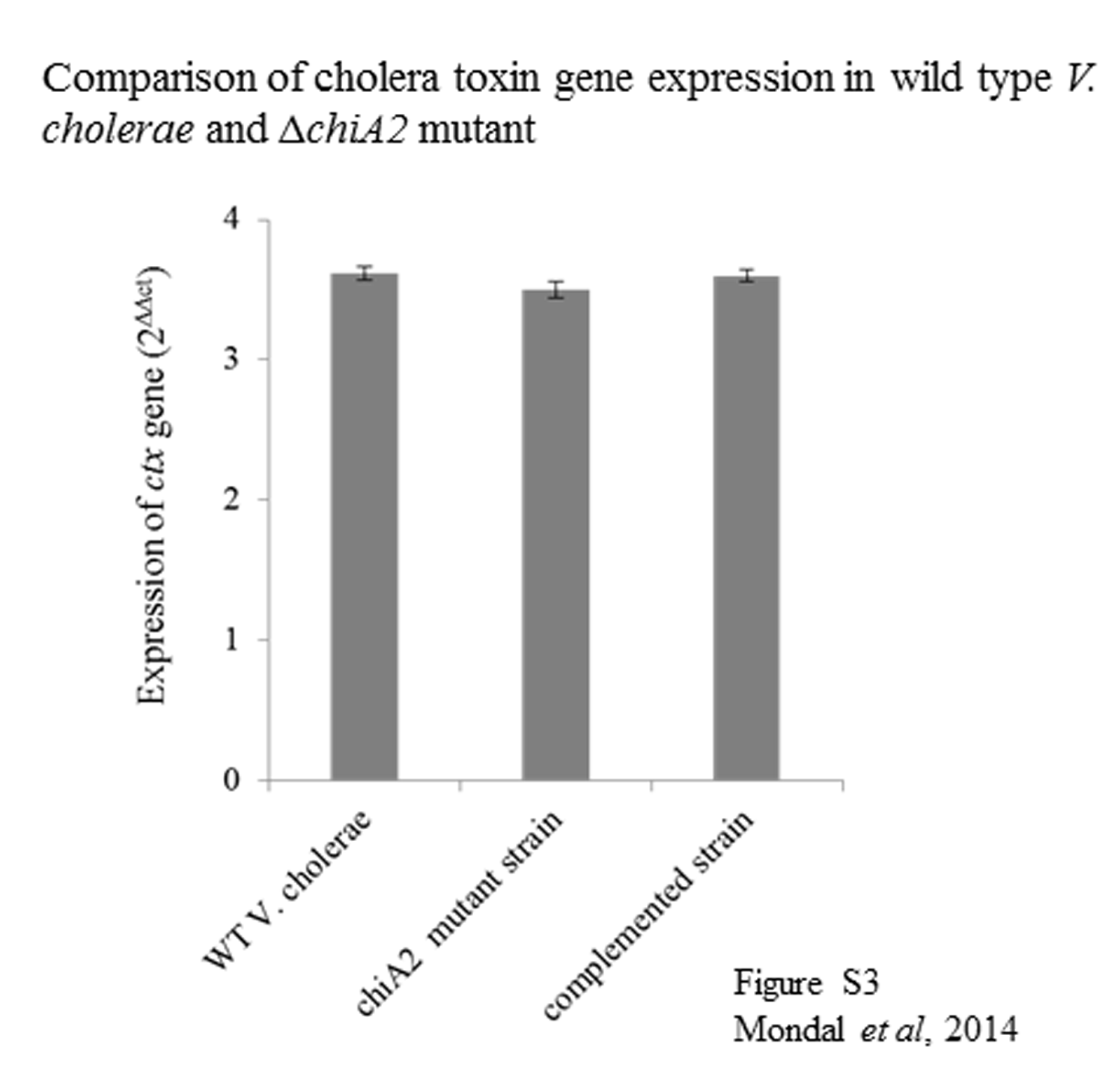

Supplement: Figure S3 — Comparison of cholera toxin gene expression in wild type V. cholerae and Δ chiA2 mutant. This figure shows comparative graphical representation of ctx gene expression in wild type V. cholerae, ΔchiA2 mutant and the complemented strain. The ctx expression was measured quantitatively by real time PCR using Power SYBR Green PCR master Mix in a 7500 real time PCR detection system. (TIF) [file pone.0103119.s003.tif]

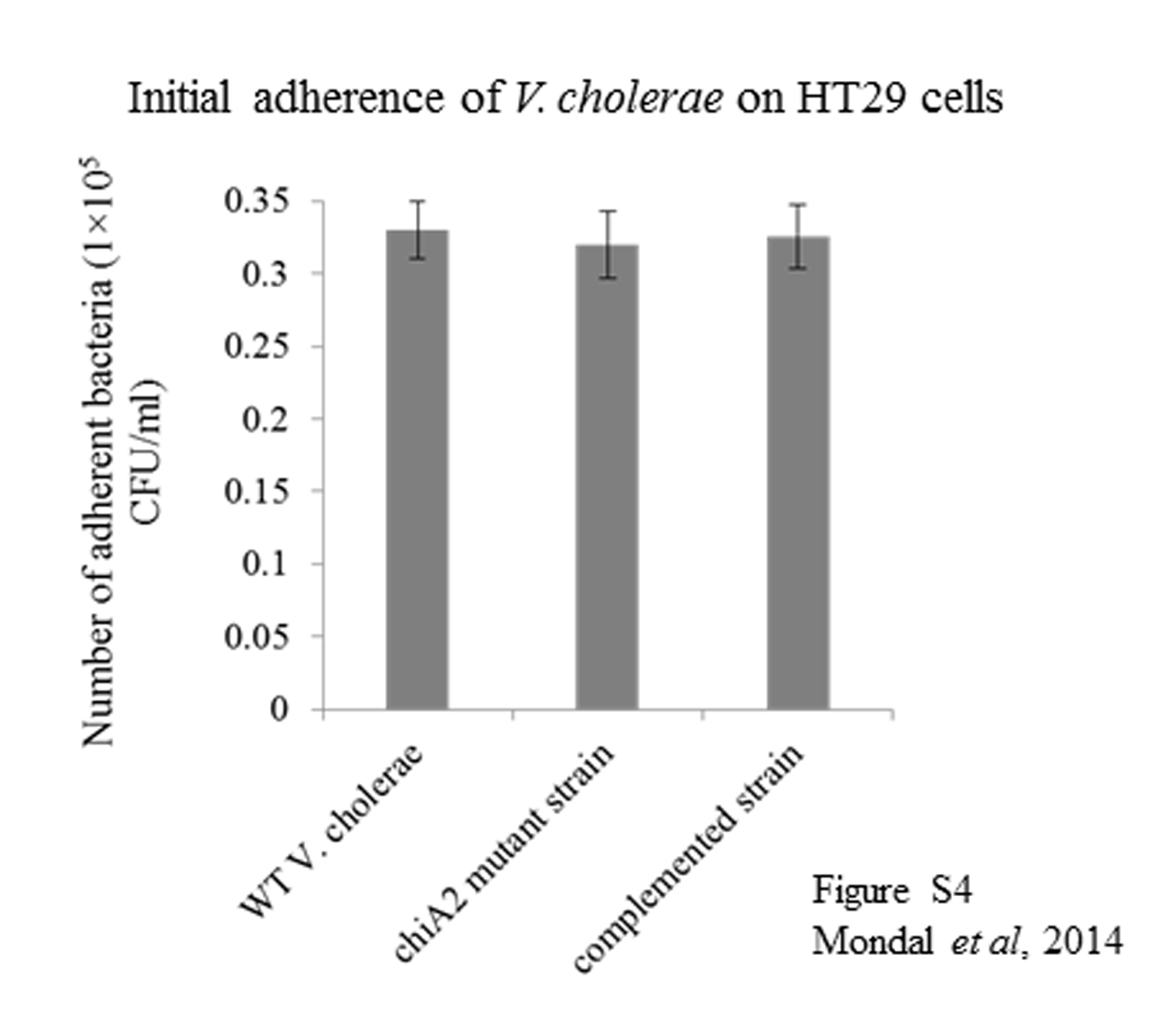

Supplement: Figure S4 — Initial adherence of V. cholerae on HT29 cells. This figure shows the graphical representation of the comparative study of initial adherence of wild type V. cholerae, ΔchiA2 mutant and the complemented strain on HT29 human intestinal epithelial cells. The bound bacteria were enumerated by plate count method using TCBS agar plates. (TIF) [file pone.0103119.s004.tif]
