# Supplementary material for: The Vibrio cholerae Extracellular Chitinase ChiA2 Is Important for Survival and Pathogenesis in the Host Intestine
Source: PLoS One. 2014 Sep 22;9(9):e103119. doi: 10.1371/journal.pone.0103119 (PMC4170974; doi:10.1371/journal.pone.0103119)
Supplement: File S1 — Elaborated Methodology. (DOC) [file pone.0103119.s005.doc]

**Elaborated Methodology**

**Generation of the *ΔchiA2 mutant* strain and its complement**

The *ΔchiA2* mutant was constructed as described previously [16]. In brief, 500-bp fragment upstream and downstream of *chiA2* were PCR-amplified using the primer pairs *chiA2* Ext F (A) and *chiA2* Fus R (B); *chiA2* Fus F (C) and *chiA2* Ext R (D) (Table 1). The amplicons were fused and amplified with the primer pair A and D to generate a 1000 base pairs amplicon with an in-frame deletion of 2331 base pairs region of *chiA2* gene. The amplicon was cloned into the suicide vector pCVD442 [16]. The resultant chimeric plasmid was transformed and maintained in *E. coli* SM10λ*pir* [17] and finally mobilized conjugally from *E. coli* SM10λ*pir* to *V. cholerae* N16961. The transconjugants were selected in ampicillin (100μg/ml) and Streptomycin (100μg/ml) double antibiotic Luria Bertani (LB) agar plates. Lastly, the transconjugants were grown in sucrose selection media (1% tryptone, 0.5% yeast extract, 10% sucrose and 1.5% agar) [16] to identify the clones where pCVD442 plasmid loss has occurred. The in-frame deletion of *chiA2* was confirmed by PCR using *chiA2* external primers (Table 1) and nucleotide sequencing using primers A and D (Table 1). PCR confirmation using external primers showed only 219 bps amplicon for Δ*chiA2* mutant instead of 2.55 kb amplicon (wild type). Deactivation of *chiA2* was confirmed by chitinase activity of the culture supernatant of the *ΔchiA2* mutant with respect to the chitinase activity of the culture supernatant of wild type *V. cholerae*. The Δ*chiA2* mutant was complemented with *pchiA2* plasmid to generate a complemented strain following a previously described procedure [16]. *pchiA2* was generated by cloning the *chiA2* gene in pBAD-TOPO TA expression vector (Invitrogen, Carlsbad, CA) along with its signal sequence using the primers mentioned in Table1

**Purification of wild type ChiA2**

*V. cholerae* cells were grown with shaking at 30ºC, in chitin supplemented alkaline sea-water media (pH 8) with 300 mM salt concentration. After 24 h the cells were harvested by centrifugation at 8000 ×*g*. The culture supernatant was collected and concentrated using Amicon concentrator. The wild type ChiA2 was purified from the concentrated culture supernatant by gel filtration chromatography. For gel filtration chromatography G-200 sephadex matrix was used as column material. The whole chromatographic method was performed by BioLogic Duo Flow Chromatographic system (Bio-Rad, Hercules, CA). The purified ChiA2 was collected in different fraction and the purity was checked by SDS-PAGE (please see the File S2 Fig S1). The presence of ChiA2 was confirmed by the immunoblotting method. The fraction with pure ChiA2 was used for further experiments.

**Purification of rabbit intestinal mucin**

Mucin from rabbit intestine was purified by a previously published method [18]. Mucin was extracted from the intestinal scrapings by dissolving it in phosphate buffer saline (PBS) (pH 7.4) containing 6M guanidine hydrochloride, 1 mM phenylmethylsulphonylfluoride (PMSF), 5 mM ethylenediaminetetraacetic acid (EDTA), 10 mM benzamidine, 5 mM N-ethylmaleimide and 0.02% sodium azide. The final concentration of guanidine hydrochloride was adjusted by using PBS and solid dithiothreitol was added to a final concentration of 10 mM. It was incubated for 5 h at 37°C. After incubation iodoacetamide was added to 25 mM and incubated overnight in the dark at room temperature. It was then centrifuged at 45,000 ×*g* for 1 h at 4°C to remove the insoluble fractions. The supernatant containing crude intestinal mucus was extensively dialyzed first against water and then against PBS to remove the chemicals and free carbohydrates. It was finally lyophilized. The presence of carbohydrate and the protein were determined using the anthrone method and modified Folin- Lowry method respectively.

**Chitinase activity assay**

The N-acetylglucosamine concentration in the reaction mixture and the chitinase activity were determined by previously established Di-Nitrosalicylic acid (DNS) method [19]. This method tests the free carbonyl groups in the reducing sugars. Chitinase activity was assayed here by estimating reducing sugars. For the assay, varied concentration of purified rabbit mucin [18] and porcine mucin (SIGMA, St. Louis, MO) (0.5 mg/ml, 1mg/ml, 1.5 mg/ml, 2 mg/ml, 2.5 mg/ml and 3 mg/ml) were incubated with 50 μg/ml purified ChiA2 in phosphate buffer pH 7.4 for 3 h at 37°C. In each case the control was done by using heat inactivated enzyme instead of active ChiA2. The reaction was stopped by adding DNS solution. The mixture was boiled at 100°C for 10 min and cooled by keeping it in ice immediately after boiling. The amount of reducing sugar was estimated by measuring the OD at 540 nm.

The amount of reducing sugar was calculated from a previously prepared standard curve. The specific activity of the enzyme was calculated by measuring the amount of GlcNAc produced in μmole /mg of protein/ min. The velocity of each reaction was calculated by measuring the amount of GlcNAc produced in μmole/ ml of reaction mixture/ min. A Lineweaver Burk plot was generated by plotting inverse of different substrate concentrations (mentioned before) in X-axis and inverse of the velocity of the reaction for each substrate concentration in Y-axis. The K_m_ and V_max_ were calculated from the equation y = 0.004x + 0.0026 (y = mx + c) which was generated from the graph. The K_cat_ was calculated from the equation K_cat_ = V_max_/ total enzyme. The K_m_/K_cat_ of the reaction kinetics was also calculated.

**HPLC analyses of the end products of ChiA2 treated mucin**

HPLC analyses were done using a 4.6 nm×250, 5 µm Zorbax Carbohydrate Analysis Column (Agilent Technologies, Santa Clara, CA) connected to a Shimadzu Prominence 20A, HPLC system. LC solutions (Shimatzu, Kyoto, Japan) program was used to control, calibrate and analyze the HPLC data.

In 20 mM phosphate buffer pH 7.4, 1mg/ml of purified rabbit mucin was incubated with 50μg/ml purified ChiA2 at 37ºC for 3 h. The reaction was terminated by 10% trichloroacetic acid (HIMEDIA, Mumbai, India) (v/v). Eighty microliter of the terminated reaction mixtures were injected to the HPLC Column operated at 40ºC. The oligosaccharides were eluted from the column by using a gradient of 60-80% acetonitrile and water. The eluted fraction was passed immediately by LC PDA detection system to detect the components of the eluted fraction. The retention time of each oligosaccharide was compared with commercially available standards.

**Generation of *V. cholerae* growth curve**

Overnight cultures of the wild type *V. cholerae*, Δ*chiA2* mutant and the complemented strain in LB broth were centrifuged and washed with PBS. The cultures were diluted to 1:10,000 and mixed in 1:4 ratios with either M9 minimal media (BD Difco, Sparks, MD) supplemented with mucin or fresh LB broth. The cultures were grown for 72 h at 37°C with shaking. The viable cells were enumerated by plating the cultures at different time points (like 2h, 4h, 8h, 24h, 30h, 48h and 72h) on thiosulfate-citrate-bile-salts-sucrose (TCBS) agar plate followed by colony count.

***In vitro* survival assay**

The intestinal epithelial HT29 cells (National Center for Cell science, Pune, India) were cultured in Dulbecco’s Modified Eagle’s Medium (DMEM), supplemented with 10% fetal bovine serum (FBS) (HIMEDIA, Mumbai, India), 1% non-essential amino acid and 1% Penicillin /Streptomycin mixture at 37°C under 5% CO_2_ in a humidified CO_2_ incubator. The HT29 cells were cultured upto 80% confluency. Before the experiment, the cells were kept in DMEM containing 0.5% FBS for 18 h for starvation. Serum-starved HT29 cells were then infected with different dilutions (1×10^5^, 1×10^6^, 1×10^7^ and 1×10^8^ CFU/ml) of *V. cholerae*, Δ*chiA2* mutant and complemented strain. The culture plates were vortexed to release the bound bacteria 18 h post infection, washed with phosphate buffered saline and plated on TCBS agar plate to enumerate the survived bound and unbound *V. cholerae*. Untreated HT-29 cell culture media was used as a negative control.
